# Investigation of *Babesia* spp. and *Theileria* spp. in ticks from Western China and identification of a novel genotype of *Babesia caballi*

**DOI:** 10.1186/s12917-024-04171-z

**Published:** 2024-07-08

**Authors:** Bing Zhang, Niuniu Zhang, Chunyan Gao, Mengyun Liu, Runda Jie, Miao Lu, Yanran Ma, Fanming Meng, Jingjing Huang, Xiao Wang, Kun Li

**Affiliations:** 1 Key Laboratory of Forensic Medicine, Institute of Medical Sciences, School of Basic Medical Sciences, Xinjiang Medical University, Urumqi City, 830011 China; 2 Xinjiang Key Laboratory of Molecular Biology for Endemic Diseases, School of Basic Medical Sciences, Xinjiang Medical University, Urumqi City, 830011 China; 3 Yanjing Medical College, Capital Medical University, Shunyi District, Beijing City, 101300 China; 4 Xinjiang 474 Hospital, China RongTong Medical Healthcare Group CO. LTD, Xinjiang Uygur Autonomous Region, Urumqi City, 830011 China; 5 National Institute for Communicable Disease Control and Prevention, Chinese Center for Disease Control and Prevention, Changping Liuzi 5, Beijing, 102206 China

**Keywords:** *Babesia caballi*, *Hyalomma asiaticum*, *Rhipicephalus microplus*, Novel genotype, Western China

## Abstract

*Babesia* spp. and *Theileria* spp. are tick-borne protozoan parasites with veterinary importance. In China, epidemiological and genetic investigations on many *Babesia* and *Theileria* species were still absent in many areas and many tick species. From Aug 2021 to May 2023, 645 ticks were collected from the body surface of domestic animals (camels, goats, sheep, and cattle) using tweezers in seven counties in three provinces including Xinjiang (Qitai, Mulei, Hutubi, and Shihezi counties), Chongqing (Youyang and Yunyang counties), and Qinghai (Huangzhong county). Three tick species were morphologically and molecularly identified (334 *Hyalomma asiaticum* from Xinjiang, 245 *Rhipicephalus microplus* from Chongqing, and 66 *Haemaphysalis qinghaiensis* from Qinghai). A total of three *Babesia* species and two *Theileria* species were detected targeting the 18S gene. The *COI* and *cytb* sequences were also recovered from *Babesia* strains for further identification. In *R. microplus* from Chongqing, *Babesia bigemina*, the agent of bovine babesiosis, was detected. Notably, in *H. asiaticum* ticks from Xinjiang, a putative novel genotype of *Babesia caballi* was identified (0.90%, 3/334), whose *COI* and *cytb* genes have as low as 85.82% and 90.64–90.91% nucleotide identities to currently available sequences. It is noteworthy whether the sequence differences of its *cytb* contribute to the drug resistance of this variant due to the involvement of *cytb* in the drug resistance of *Babesia*. In addition, *Theileria orientalis* and *Theileria annulata* were detected in *R. microplus* from Chongqing (12.20%, 31/245) and *H. asiaticum* from Xinjiang (1.50%, 5/334), respectively. These results suggest that these protozoan parasites may be circulating in domestic animals in these areas. The pathogenicity of the novel genotype of *B. caballi* also warrants further investigation.

**Supplementary Information:**

The online version contains supplementary material available at 10.1186/s12917-024-04171-z.

## Introduction

*Babesia* and *Theileria* spp. belonging to the phylum Apicomplexa are protozoan parasites with veterinary importance. They are distributed worldwide and cause significant economic losses in husbandry, especially in the tropic and subtropic areas [1]. To date, more than 100 species of *Babesia* and *Theileria* have been recorded worldwide, many of which are pathogens for humans and domestic animals. For example, *Babesia ovis*, *B. motasi*, *B. crassa*, *B. foliata*, *B. taylori* are common agents of ovine babesiosis [2], while *B. caballi*, *Theileria equi*, and *T. haneyi* usually infect equines [3]. They are also frequently detected in wild animals. For example, *Babesia* spp. have been detected in *Alpine chamois*, roe deer, red deer, and wild boar, while *Theileria* spp. were identified in foxes, wild boar, and red deer [4]. These parasites parasitize in erythrocytes and lymphocytes of their animal hosts, resulting in loss of body weight, milk output, and even animal death [5]. Some of them are also considered agents of emerging zoonosis, such as *B. divergens* and *B. microti* [6].

Ticks, a group of obligatory hematophagous arthropods usually infest domestic and wild animals, are the major vectors of *Babesia* and *Theileria* spp. To date, more than 21 tick species, mainly hard ticks, have been reported to transmit these parasites [7]. Meanwhile, asymptomatic animals usually act as their reservoirs. In China, at least 16 *Babesia* species (*B. bigemina*, *B. bovis*, *B. major*, *B. motasi*, *B. ovis*, *B. divergens*, *B. microti*, *B. perroncitoi*, *B. trautmanni*, *B. orientalis*, *B. gibsoni*, *B. canis*, *B. occultans*, *B. venatorum*, *B. ovata*, and *B. caballi*) and 6 *Theileria* species (*T. ovis*, *T. orientalis*, *T. sinensis*, *T. uilenbergi*, *T. luwenshuni*, and *T. annulata*) have been detected in various ticks and animals [8]. However, compared to many other tick-borne pathogens (*Rickettsia*, *Anaplasma*, *Coxiella*, *Borrelia*, etc.), these tick-borne protozoan parasites are still largely neglected and unstudied in many areas and many tick species in China. Genetic investigations were also absent for many *Babesia* and *Theileria* species. In this study, we collected three tick species from three provinces in Western China, and investigated the genetic diversity of *Babesia* spp. and *Theileria* spp. in them.

## Materials and methods

### Sample collection, DNA extraction, and identification of the ticks

From Aug 2021 to May 2023, a total of 645 ticks were collected from the body surface of domestic animals in seven locations (counties, districts, or county-level cities) in three provinces: Qitai, Mulei, Hutubi, and Shihezi counties in Xinjiang Uygur Autonomous Region (from cattle, goats, and camels); Yunyang and Youyang counties in Chongqing Municipality (from cattle); Huangzhong county in Qinghai Province (from goats) (Fig. S1). Ticks were carefully removed from the body surface of domestic animals (camels, goats, sheep, and cattle) using tweezers. Probability sampling was used in this study. First of all, the collected ticks were morphologically identified into species according to their morphological features including the capitula, legs, anal groove, etc. [9, 10]. After washing twice with PBS, the ticks were subjected to DNA extraction using the TIANamp genomic DNA Extraction Kit (TIANGEN company), and then the *COI* gene sequences were amplified for molecular confirmation of the tick species (Primers shown in reference [11]).

### Detection and identification of the *Babesia* spp. and *Theileria* spp.

All the 645 DNA samples were screened for *Babesia* spp. and *Theileria* spp. using primers amplifying a conserved region of the 18S gene, yielding an approximately 600–650 bp product. Primer pairs of New-Babesia-F/New-Babesia-R1 and New-Babesia-F/New-Babesia-R2 were designed in this study (our lab designed all the primers and the sequences are shown in Table S1). The PCR conditions of first round PCR reaction: 94 °C for 3 min, 40 cycles of denaturation at 94 °C for 40 s, annealing at 50 °C for 40 s, and extension at 72 °C for 60 s, with a final extension at 72 °C for 8 min. The PCR conditions of second round PCR reaction: 94 °C for 3 min, 40 cycles of denaturation at 94 °C for 40 s, annealing at 48 °C for 40 s, and extension at 72 °C for 45 s, with a final extension at 72 °C for 8 min. The PCR products were then subjected to sequencing in Sangon Company. The obtained sequences were then aligned with reference sequences in the GenBank Database by BLASTN (https://blast.ncbi.nlm.nih.gov/Blast.cgi) to determine their genus and species initially.

For further identification of the detected *Babesia* strains, the mitochondrial *cytb* (cytochrome b) and *COI* (cytochrome oxidase subunit I) genes were PCR amplified from randomly selected samples which are positive for each *Babesia* species. The primers used were shown in the reference [12]. The PCR conditions of *cytb*: 94 °C for 5 min, 40 cycles of denaturation at 94 °C for 40 s, annealing at 55 °C for 40 s, and extension at 72 °C for 90 s, with a final extension at 72 °C for 8 min. The PCR conditions of *COI*: 94 °C for 5 min, 40 cycles of denaturation at 94 °C for 40 s, annealing at 60 °C for 40 s, and extension at 72 °C for 70 s, with a final extension at 72 °C for 8 min.

### Genetic and phylogenetic analysis of the sequences

All the recovered nucleotide sequences were compared with references in the GenBank Database analyzed by BLASTN (https://blast.ncbi.nlm.nih.gov/Blast.cgi). Most, if not all, formally validated *Babesia* and *Theileria* species were selected as reference sequences. Along with reference sequences, the sequences were manually aligned by the ClustalW method in the MEGA program. Maximum Likelihood (ML) trees were reconstructed in the GTR model using PhyML v3.0 [13]. The substitution model test was performed to select the best-fit phylogenetic model. The confidence values for each branch of the trees were determined by bootstrap analysis with 100 repetitions, and the confidence values larger than 70 were considered adequate. All the phylogenetic trees were mid-point rooted.

## Results

### Determination of the tick species

All the 334 ticks from Xinjiang were identified to be *Hyalomma asiaticum*. Meanwhile, the 245 ticks from Chongqing and 66 ticks from Qinghai were identified to be *Rhipicephalus microplus* and *Haemaphysalis qinghaiensis*, respectively. The detailed information of ticks is shown in Table 1. All the tick species were molecularly confirmed after morphological identification. The *COI* sequences were all > 99% identical to those in the GenBank Database.

**Table 1:** Prevalence of *Babesia* spp. and *Theileria* spp. in ticks from three provinces of China

	**Xinjiang**				**Qinghai**	**Chongqing**	
	Qitai county (cattle)	Mulei county (camels)	Hutubi county (goats)	Shihezi city (goats)	Xining city (goats)	Youyang county (cattle)	Yunyang county (cattle)
	*Hy. asiaticum*	*Hy. asiaticum*	*Hy. asiaticum*	*Hy. asiaticum*	*Ha. qinghaiensis*	*Rh. microplus*	*Rh. microplus*
*Babesia bigemina*	0/120 (0.00%)	0/72 (0.00%)	0/46 (0.00%)	0/96 (0.00%)	0/66 (0.00%)	2/156 (1.28%)	0/89 (0.00%)
*Babesia caballi*	2/120 (1.67%)	1/72 (1.39%)	0/46 (0.00%)	0/96 (0.00%)	0/66 (0.00%)	0/156 (0.00%)	0/89 (0.00%)
*Babesia* sp.	11/120 (9.17%)	1/72 (1.39%)	1/46 (2.17%)	8/96 (8.33%)	0/66 (0.00%)	0/156 (0.00%)	0/89 (0.00%)
*Theileria orientalis*	0/120 (0.00%)	0/72 (0.00%)	0/46 (0.00%)	0/96 (0.00%)	0/66 (0.00%)	31/156 (19.87%)	0/89 (0.00%)
*Theileria annulata*	5/120 (4.17%)	0/72 (0.00%)	0/46 (0.00%)	0/96 (0.00%)	0/66 (0.00%)	0/156 (0.00%)	0/89 (0.00%)

### Detection and identification of *Babesia* spp.

**Fig. 1:**
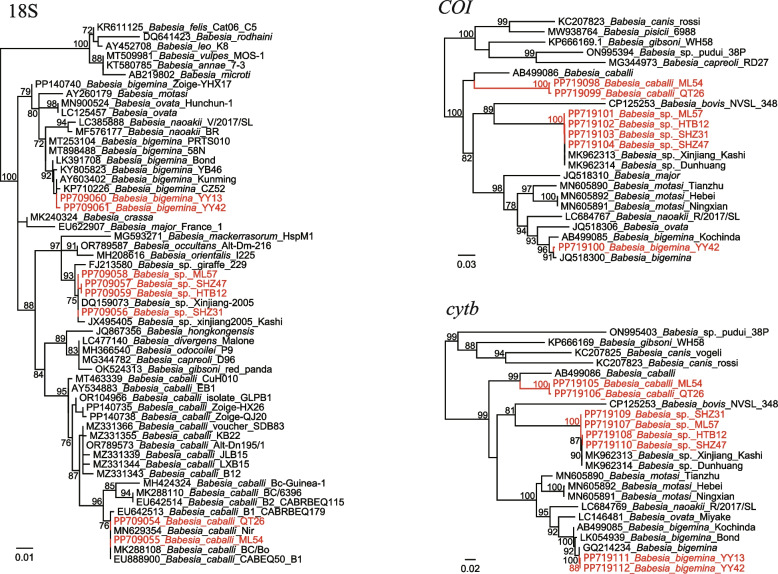
Phylogenetic trees based on the nucleotide sequences of 18S rRNA (Size: 538–548 bp), *COI* (Size: 977 bp), and *cytb* (Size: 1132 bp) genes of *Babesia* strains. Maximum Likelihood (ML) trees were reconstructed in the GTR model using PhyML v3.0

PCR detection and sequence analysis indicated three *Babesia* species were identified: *B. bigemina*, *B. caballi*, and *Babesia* sp. Of those, *B. bigemina* was only detected in *R. microplus* ticks from Youyang County of Chongqing, with a positive rate of 1.28% (2/156). BLASTN (https://blast.ncbi.nlm.nih.gov/Blast.cgi) shows that the 18S (PP709054-PP709061), *COI* (PP719098-PP719104), and *cytb* (PP719105-PP719112) genes are 99.81–100%, 100%, and 99.73% to reference strains in the GenBank Database (Accession numbers for 18S: AY603402.1. *COI*: PP719100.1. *cytb*: GQ214234.1). An unnamed *Babesia* species was detected in *H. asiaticum* ticks from three counties (Shihezi, Mulei, and Hutubi) in Xinjiang, with positive rates of 8.33%, 1.39%, and 9.17% (Table 1). Genetic and phylogenetic analysis indicated that these strains were all highly homologous from each other, and all their 18S, *COI*, and *cytb* sequences are closely related to those of strains previously identified in sheep from Xinjiang (Accession numbers for 18S: DQ159073.1, JX495405.1. *COI*: MK962313.1, MK962314.1. *cytb*: MK962313.1, MK962314.1) (Fig. 1).

Notably, a *B. caballi* variant was identified in *H. asiaticum* ticks from Qitai (1.67%, 2/120) and Mulei (1.39%, 1/72) counties in Xinjiang. Although its 18S gene showed 100% identity to some previously reported *B. caballi* strains, the *COI* and *cytb* genes have as low as 85.82% and 90.64–90.91% nucleotide identities to the *B. caballi* strain from Japan (Accession numbers for the *COI* and *cytb* genes: AB499086.1). In the phylogenetic trees, they also form relatively independent clades (Fig. 1). Therefore, we suppose that they represent a previously uncharacterized novel genotype of *B. caballi*.

No *H. qinghaiensis* ticks from Qinghai Province were positive for *Babesia*. All the obtained sequences have been uploaded to the GenBank Database (Table S2).

### Detection and identification of *Theileria* spp.

**Fig. 2:**
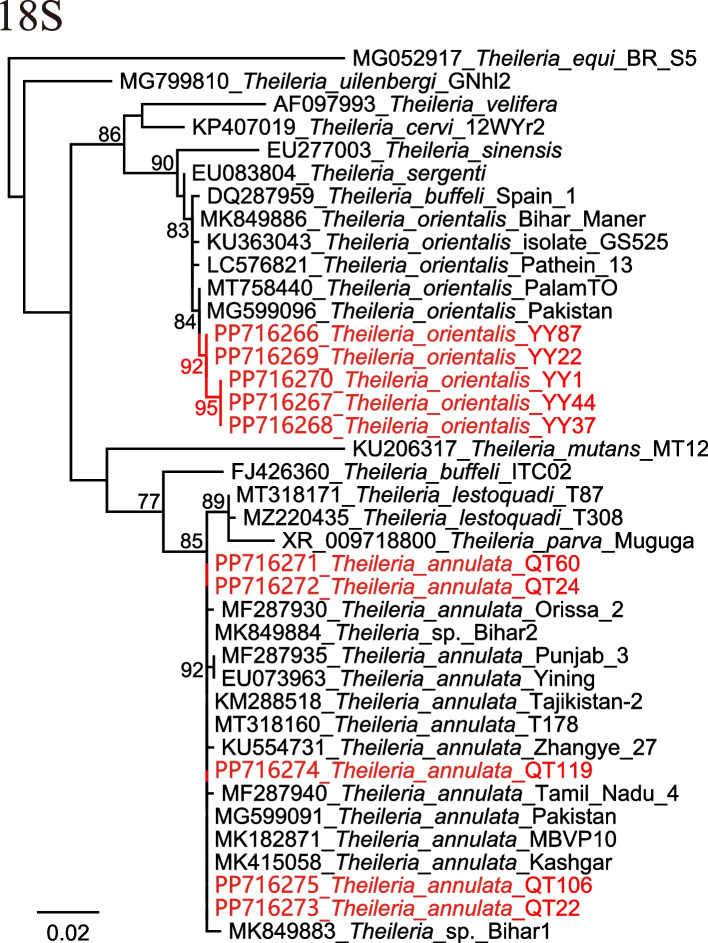
Phylogenetic trees based on the nucleotide sequences of 18S rRNA (Size: 588–591 bp) gene of *Theileria* strains. Maximum Likelihood (ML) trees were reconstructed in the GTR model using PhyML v3.0

Two *Theileria* species were detected in the ticks. In *R. microplus* ticks from Youyang County of Chongqing, *T. orientalis* was detected with a high prevalence (19.87%, 31/156). BLASTN (https://blast.ncbi.nlm.nih.gov/Blast.cgi) shows that the 18S sequences (PP716266-PP716272) have 99.49–99.83% homology to *T. orientalis* strains from India and Pakistan (Accession numbers: MT758440.1 and MG599096.1) (Fig. 2). In *H. asiaticum* ticks from Qitai County of Xinjiang, *Theileria annulata* was identified with a positive rate of 4.17% (5/120). All their 18S sequences are 100% identical to those of previously reported *T. annulata* strains from Pakistan (Accession numbers: MG599091.1 and MT318160.1), Tajikistan (Accession number: KM288518.1), Iraq (Accession number: MK182871.1), India (Accession number: MK849884.1), and China (Accession number: MK415058.1).

No *H. qinghaiensis* ticks from Qinghai Province were positive for *Theileria*.

## Discussion

Among the three detected *Babesia* species, *B. bigemina* is a worldwide distributed agent of bovine babesiosis. It was also reported to infect other animals such as goats [14]. In China, *B. bigemina* has been reported in ticks (mainly *R. microplus*) and domestic animals from many provinces including Gansu, Yunnan, Guangxi, Chongqing, Shandong, Liaoning, Henan, Hubei, and Xinjiang, suggesting its wide geographic distribution [15]. Actually, it is considered one of the most prevalent agents causing bovine babesiosis in China [16]. In this study, *B. bigemina* was detected in *R. microplus* ticks collected from cattle in Chongqing. Although the positive rate is low, it suggests that *B. bigemina* may be circulating in cattle in this area.

An unnamed *Babesia* species was identified in *H. asiaticum* from Xinjiang, with all its sequences (18S, *COI*, and *cytb*) highly homologous to *Babesia* strains previously identified in sheep from Xinjiang (DQ159073.1, MK962313.1, MK962313.1). Interestingly, all *H. asiaticum* ticks positive for this *Babesia* were removed from cattle, camels, and goats. This suggests two possibilities: First, the *Babesia* sp. may be from the blood meal of infected cattle, camels, and goats. Namely, it may be pathogenic to these domestic animals. Second, *H. asiaticum* ticks might be the vector of this *Babesia* and play a role in its transmission or maintenance. In future studies, the pathogenicity of this *Babesia* to domestic animals still warrants further investigation.

It is out of our expectation that a novel genotype of *B. caballi* was identified in *H. asiaticum* ticks from Xinjiang. *Babesia caballi* is the agent of equine piroplasmosis, an economically important tick-borne disease worldwide [17]. The infection of *B. caballi* in horses usually causes anemia, hemoglobinuria, fever, abdominal inflammation, weakening, etc. [18]. In China, some molecular and serological investigations targeting *B. caballi* have been carried out, reporting its presence in multiple provinces including Xinjiang, Jilin, and Gansu [19–21]. Although reports on *B. caballi* is common, further analysis such as genotyping has been rare. Most studies are preliminary and very few references were available except for the 18S gene. In this study, we identified a novel genotype of *B. caballi*, whose *cytb* and *COI* sequences show low homologies to existing sequences of *B. caballi*. The phylogenetic trees also show remarkable distances with other *B. caballi* strains and currently known *Babesia* species. The amino acid sequences of *cytb* and *COI* genes were also analyzed. The *cytb* sequences have highest 97.80% identity to *B.caballi* from Japan (GIX66475.1), while the *COI* sequences show 91.39% identity to reference *B. caballi* strains (BAI66165.1). This result confirmed that it represents a novel genotype. We suppose that the sequence difference may be due to the absence of enough reference sequences in the GenBank Database. As more sequences of *B. caballi* will be obtained in the future, we believe more genotypes may be identified. Besides, as was previously reported, the *cytb* gene is involved in the drug resistance of *Babesia* [22–24]. It is noteworthy that the sequence differences contribute to the drug resistance of this variant.

Two *Theileria* species were detected in this study. Of those, *T. annulata* is the most pathogenic species in cattle causing tropical theileriosis [25], while *T. orientalis* is the most prevalent *Theileria* species in China [26]. Both these two species are of economic importance in husbandry and veterinary. Ticks were considered vectors of *Theileria*. In this study, *T. annulata* was only detected in *H. asiaticum*, and *T. orientalis* was only identified in *R. microplus* ticks. However, the vector roles of both *H. asiaticum* and *R. microplus* are still to be determined, despite the frequent reports of *Theileria* in them [27–31]. For *T. orientalis*, although its DNA has been detected in eggs of *R. microplus* tick [32], suggesting the existence of transovarial transmission, a study performed by Ghafar et al. [33] indicated that *R. microplus* fails to transmit *T. orientalis*. Notably, in this study, all the *Theileria*-positive ticks were fully engorged and were collected from cattle, indicating that the *Theileria* DNA might be from cattle blood. Namely, *T. annulata* and *T. orientalis* may be circulating in cattle in these areas. Furthermore, the phylogenetic tree also shows that the detected *T. orientalis* strains were divided into two clades (Fig. 2), suggesting its genetic diversity in this area. The characteristics of other genes, and their relational virulence and drug resistance still warrant further investigations.

## Conclusion

A total of three *Babesia* species (*B. bigemina*, *B. caballi*, and *Babesia* sp.) and two *Theileria* species (*T. orientalis and T. annulata*) were detected in 645 ticks representing three species (*H. asiaticum*, *R. microplus*, and *H. qinghaiensis*) from western China. Of those, a novel genotype of *B. caballi* was identified. These results proved the wide circulation and remarkable genetic diversity of *Babesia* and *Theileria* in China.

### Supplementary Information

Supplementary Material 1.

Supplementary Material 2.

Supplementary Material 3.

## Data Availability

All sequence data have been uploaded to the GenBank Database and the accession numbers are available in Table S2 (Accession Numbers: PP709054-PP709061, PP716266-PP716275, PP719098-PP719112).
