# Supplementary material for: Investigation of Babesia spp. and Theileria spp. in ticks from Western China and identification of a novel genotype of Babesia caballi
Source: BMC Vet Res. 2024 Jul 8;20:302. doi: 10.1186/s12917-024-04171-z (PMC11229187; doi:10.1186/s12917-024-04171-z)
Supplement: Supplementary file 1 — Supplementary Material 1. [file 12917_2024_4171_MOESM1_ESM.doc]

Table S1. The primers used to amplify the 18S gene from *Babesia* spp. and *Theileria* spp. by hemi-nested PCR.

| Primer | Cycle | Sequence | Anticipated amplicon length | Annealing temperature |
| --- | --- | --- | --- | --- |
| New-Babesia-F | 1, 2 | 5’-GTAATTCCAGCTCCAATAGC-3’ | 600-650 bp | 50^o^C and 48^o^C |
| New-Babe-R1 | 1 | 5’-ACGGCTACCTTGTTACGACTT-3’ |  | 50^o^C |
| New-Babe-R2 | 2 | 5’-AGTAYCGRACCAGATAGCCGC-3’ |  | 48^o^C |
