# Supplementary material for: Investigation of Babesia spp. and Theileria spp. in ticks from Western China and identification of a novel genotype of Babesia caballi
Source: BMC Vet Res. 2024 Jul 8;20:302. doi: 10.1186/s12917-024-04171-z (PMC11229187; doi:10.1186/s12917-024-04171-z)
Supplement: Supplementary file 2 — Supplementary Material 2. [file 12917_2024_4171_MOESM2_ESM.docx]

Table S2. Accession numbers of the nucleotide sequences of *Babesia* and *Theileria* strains in this study in the GenBank database.

| No. | **Gene** | **Accession Number** | ***Babesia* and *Theileria* strains** |
| --- | --- | --- | --- |
| 1 | 18S | PP709054 | *Babesia_caballi*_QT26 |
| 2 | 18S | PP709055 | *Babesia_caballi*_ML54 |
| 3 | 18S | PP709056 | *Babesia_*sp._SHZ31 |
| 4 | 18S | PP709057 | *Babesia_*sp._SHZ47 |
| 5 | 18S | PP709058 | *Babesia_*sp._ML57 |
| 6 | 18S | PP709059 | *Babesia_*sp._HTB12 |
| 7 | 18S | PP709060 | *Babesia_bigemina*_YY13 |
| 8 | 18S | PP709061 | *Babesia_bigemina*_YY42 |
| 9 | 18S | PP716266 | *Theileria_orientalis*_YY87 |
| 10 | 18S | PP716267 | *Theileria_orientalis*_YY44 |
| 11 | 18S | PP716268 | *Theileria_orientalis*_YY37 |
| 12 | 18S | PP716269 | *Theileria_orientalis*_YY22 |
| 13 | 18S | PP716270 | *Theileria_orientalis*_YY1 |
| 14 | 18S | PP716271 | *Theileria_annulata*_QT60 |
| 15 | 18S | PP716272 | *Theileria_annulata*_QT24 |
| 16 | 18S | PP716273 | *Theileria_annulata*_QT22 |
| 17 | 18S | PP716274 | *Theileria_annulata*_QT119 |
| 18 | 18S | PP716275 | *Theileria_annulata*_QT106 |
| 19 | *COI* | PP719098 | *Babesia_caballi*_ML54 |
| 20 | *COI* | PP719099 | *Babesia_caballi*_QT26 |
| 21 | *COI* | PP719100 | *Babesia_bigemina*_YY42 |
| 22 | *COI* | PP719101 | *Babesia_*sp._ML57 |
| 23 | *COI* | PP719102 | *Babesia_*sp._HTB12 |
| 24 | *COI* | PP719103 | *Babesia_*sp._SHZ31 |
| 25 | *COI* | PP719104 | *Babesia_*sp._SHZ47 |
| 26 | *cytb* | PP719105 | *Babesia_caballi*_ML54 |
| 27 | *cytb* | PP719106 | *Babesia_caballi*_QT26 |
| 28 | *cytb* | PP719107 | *Babesia_*sp._ML57 |
| 29 | *cytb* | PP719108 | *Babesia_*sp._HTB12 |
| 30 | *cytb* | PP719109 | *Babesia_*sp._SHZ31 |
| 31 | *cytb* | PP719110 | *Babesia_*sp._SHZ47 |
| 32 | *cytb* | PP719111 | *Babesia_bigemina*_YY13 |
| 33 | *cytb* | PP719112 | *Babesia_bigemina*_YY42 |
