# Supplementary figures and images for: Investigation of Babesia spp. and Theileria spp. in ticks from Western China and identification of a novel genotype of Babesia caballi
Source: BMC Vet Res. 2024 Jul 8;20:302. doi: 10.1186/s12917-024-04171-z (PMC11229187; doi:10.1186/s12917-024-04171-z)

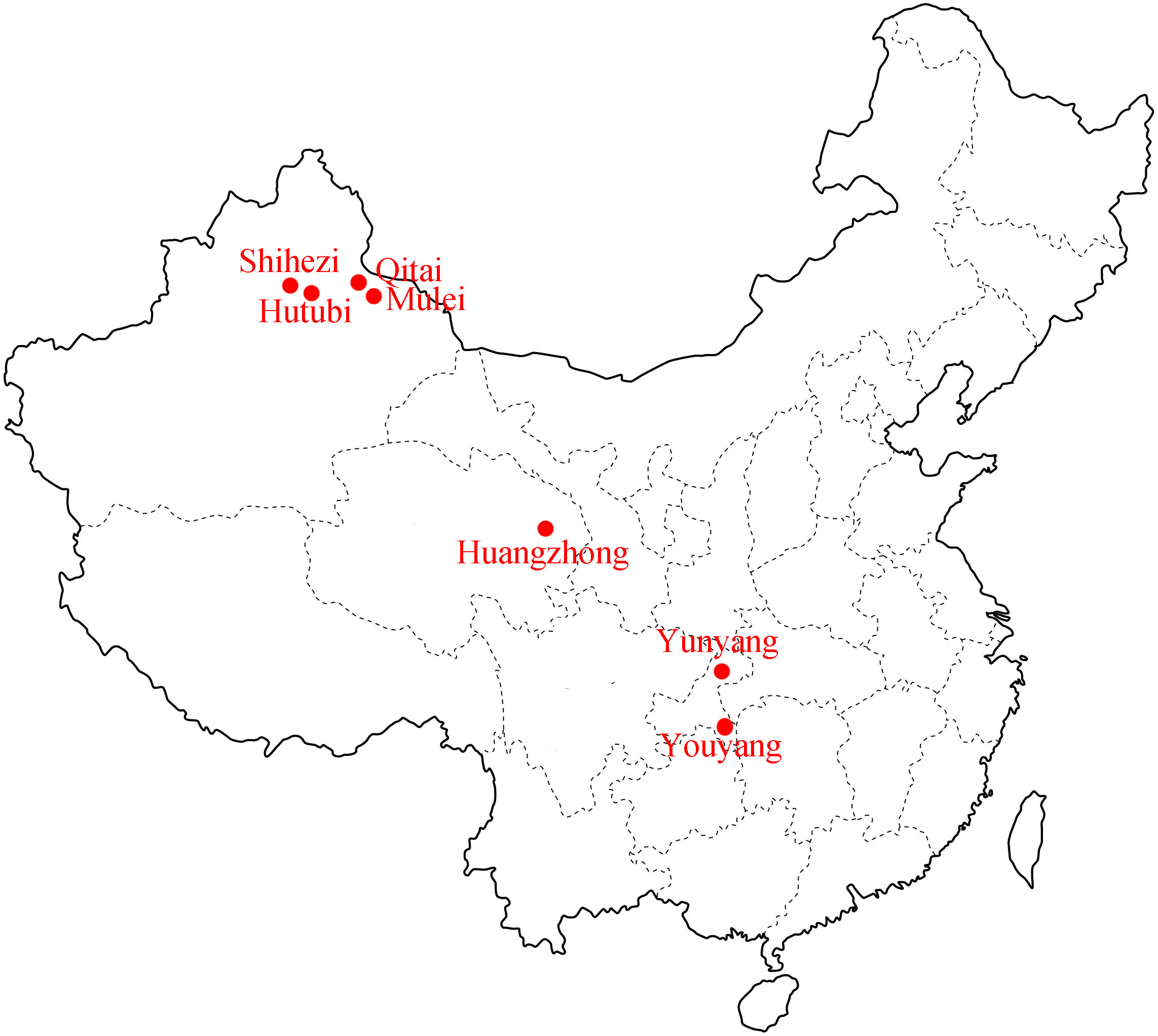

Supplement: Supplementary file 3 — Supplementary Material 3. [file 12917_2024_4171_MOESM3_ESM.jpg]
